# Migrating BioEnterics® Intragastric Balloon in a Patient Presenting With Symptoms of Intestinal Obstruction: A Case Report

**DOI:** 10.7759/cureus.36515

**Published:** 2023-03-22

**Authors:** Afnan Almuhanna, Reem Althwanay, Razan Alshehri, Buthainah A AlGarni

**Affiliations:** 1 Radiology, King Fahad University Hospital, Khobar, SAU; 2 Radiology, Imam Abdulrahman Bin Faisal University, Dammam, SAU

**Keywords:** obesity, bariatric surgery, bowel obstruction, gastroenteritis, bioenteric intragastric balloon

## Abstract

Intragastric balloons are one method of obesity treatment. We report on a 34-year-old man who presented with colicky abdominal pain with nausea and vomiting a year and eight months after intragastric balloon insertion. To evaluate the patient's symptoms, an initial abdominal X-ray was done followed by a computed tomography scan to check the condition of the intragastric balloon. In our case, the patient's pain was not due to the deflated migrating BioEnterics® intragastric balloon (BIB). Thus, no further management was done for it, and the patient was diagnosed with gastroenteritis. Despite that, regular follow-up is recommended to prevent serious complications in the future.

## Introduction

Over one billion people around the world are categorized as obese. This high rate of obesity leads to an increase in obesity-related health conditions. Crossan and Sheer found that these numbers are expected to reach an all-time high by 2030, which necessitates finding an effective method of treatment and management [1]. The management plan usually depends on the patient's BMI. According to the NIH, if a patient has a BMI above 40 or BMI between 39.9 and 35 with associated comorbidities, surgical intervention is usually recommended [2]. One of the current methods used is the insertion of an intragastric balloon, which is a minimally invasive procedure where a balloon is placed in the stomach endoscopically and then inflated with either air, saline, or methylene blue. The patient is usually followed up for six months and depending on the type of balloon, it can be removed at six months or a few months later [3]. Herein, we report a case of abdominal pain caused by a deflated migrating BioEnteric® intragastric balloon (BIB), a year and eight months after its insertion.

## Case presentation

A 34-year-old Syrian man, without any known co-morbidities, presented with severe, progressive, generalized, colicky abdominal pain that was more localized in the lower abdomen. The pain started a day before his presentation after eating at a restaurant and was accompanied by anorexia, nausea, and vomiting. The patient mentioned that he had passed flatus but was constipated during the last two days. One year and eight months prior to his presentation, he had a BIB insertion through an esophagogastroduodenoscopy. The patient is a candidate for bariatric surgery due to him suffering from morbid obesity with a BMI of 41 kg/m^2^; however, he chose to undergo BIB insertion due to it being a less invasive option. The BIB was not removed since then.

**Figure 1:**
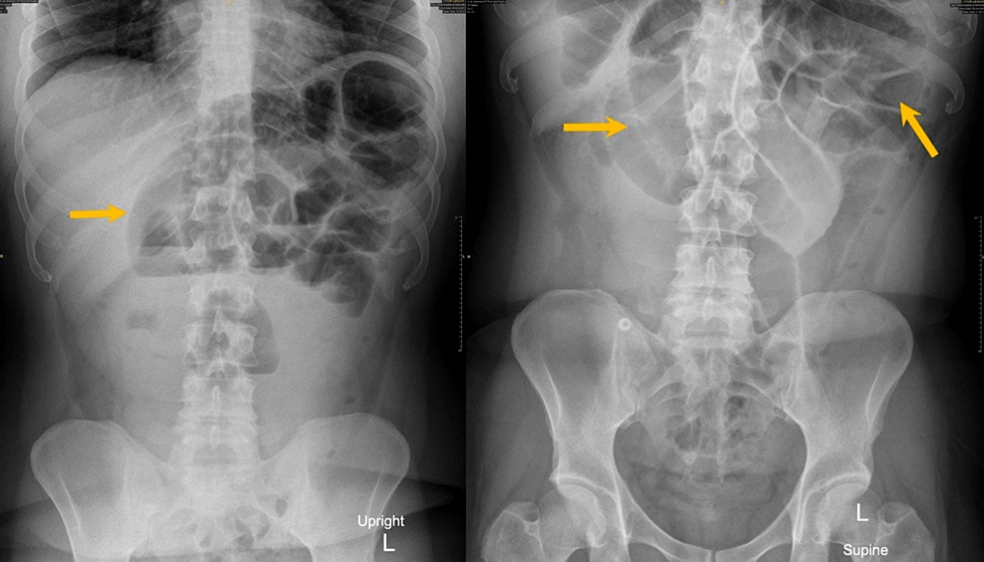
Showing an erect and supine film demonstrating dilated small bowel loops

The patient appeared to be in pain with mild signs of dehydration. On abdominal examination, there was mild epigastric tenderness but no guarding, rigidity, rebound tenderness, or distention. Bowel sounds were audible, of a low-pitched and gurgling nature. An initial abdominal X-ray was done to see if there was any evidence of intestinal obstruction and the findings were dilated small bowel loops with no air-fluid level (Figure 1).

**Figure 2:**
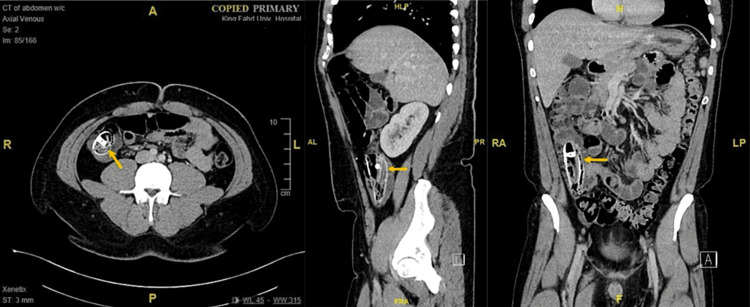
Axial, sagittal, and coronal views of a large, oblong-shaped foreign body in the cecum and ascending colon

Computed tomography of the abdomen with intravenous contrast was performed, which demonstrated a large oblong-shaped foreign body within the cecum and ascending colon. The foreign body seemed to contain a mix of metallic and plastic components that contained gas locules. The stomach appeared normal and was free of any intra-gastric balloon-like structures. These findings are highly consistent with a spontaneously deflated and migrated intra-gastric balloon. Furthermore, there were no signs of bowel obstruction and no free air or fluid in the intestine (Figure 2). 

Eventually, the patient was managed with IV fluids and analgesia, which resolved his symptoms. Moreover, due to the results of the imaging, clinical findings, and the patient's improvement with symptomatic treatment, it was concluded that the most likely cause of the symptoms was gastroenteritis and the foreign body found on the CT was an incidental finding that doesn't require urgent management. For this reason, he was discharged with instructions to come to the emergency room if the symptoms worsen and had a follow-up appointment with gastroenterology to discuss further management. The patient did not present again to the emergency department in the following months.

## Discussion

In a Lancet article, obesity was defined as a body mass index of 30 kg/m^2^ and higher. According to the latest observational data of the World Health Organization (WHO) from 2016, an estimated 650 million individuals were reported to be obese, virtually marking obesity a global, growing pandemic. Many etiologies are associated with obesity, however, most cases are associated with a sedentary lifestyle and increased caloric intake. Obesity is associated with many adverse effects on life and health, including a decrease in life expectancy and an increased risk of several cancer types and cardiovascular diseases [2,4,5].

Another article by Wolfe et al. discussed a lot of different modality options for the management of obesity [6]. These modalities range from lifestyle modifications to medications and bariatric surgeries. The consensus to recommend bariatric surgery to patients depends on multiple factors including BMI, the presence of co-morbidities, and patient preference. Adolescents and adults with a BMI of 40 and above or 35-39.9 with at least one comorbidity make the patient a candidate for bariatric surgery. In our case, the patient had a BMI of 41.1, which made him an excellent candidate for bariatric surgery. However, due to the wishes of this patient to undergo a less invasive approach, he went with BIB insertion.

Silva and Mathus-Vliegen explained that there are different methods of bariatric procedures and the selection of each depends on the patient’s characteristics [7,8]. The intragastric balloon procedure that was used in our patient is a temporary and non-invasive procedure for weight loss. It mainly works as a space-occupying device to reduce the stomach's capacity, aiding in decreasing hunger and food intake. Moreover, the balloon itself has different models, filled with either liquid or air, adjustable or non-adjustable. In our case, the patient underwent BIB placement, which promotes 5-9 BMI unit loss within six months [8].

The aforementioned article by Mathus-Vliegen described how the intragastric balloon insertion is considered a preferred method due to its non-invasive placement; however, it is still associated with some adverse outcomes such as esophagitis, intolerance, and balloon deflation and migration leading to obstruction [8]. A case report by Al Shammari et al. described a case of intragastric balloon migration where the patient presented with symptoms of intestinal obstruction two years following BIB insertion [9]. It was later found out that the balloon deflated and migrated to the ileocolic region, causing subacute intestinal obstruction, which was surgically treated using a laparoscopic approach. It was inferred from it that the importance of follow-up and adherence to the recommended period that BIB should be kept. An article by Herve et al. mentioned that the recommended period of placement for BIB is six months, after which it should be removed to reduce complications [10]. However, in our case, the patient underwent the procedure a year and eight months ago and did not remove it due to the loss of follow-up, which led to balloon deflation and migration.

## Conclusions

This case report presents a patient with a migrating BIB. The patient presented with signs and symptoms of obstruction. For this reason, an abdominal X-ray followed by a CT scan was done, which ruled out an intestinal obstruction and demonstrated a deflated and migrated balloon. Hence, the patient's symptoms were attributed to gastroenteritis and the migration of BIB was probably due to the loss of follow-up and exceeding the appropriate time frame before its removal. Thus, we conclude the importance of regular follow-ups for these patients.
